# Clinically Mild Encephalopathy with a Reversible Splenial Lesion Caused by Influenza B Virus in an Unvaccinated Child

**DOI:** 10.3390/pediatric13010009

**Published:** 2021-02-04

**Authors:** Silvia Ventresca, Claudia Guiducci, Sara Tagliani, Sara Dal Bo, Paolo Ricciardelli, Patrizia Cenni, Federico Marchetti

**Affiliations:** 1 Department of Pediatrics, S. Maria delle Croci Hospital, 48121 Ravenna, Italy; claudia.guiducci2@gmail.com (C.G.); sara.tagliani@student.unife.it (S.T.); sara.dal.bo@gmail.com (S.D.B.); paolo.ricciardelli@auslromagna.it (P.R.); federico.marchetti@auslromagna.it (F.M.); 2 Department of Medical Sciences, Pediatrics, University of Ferrara, 44121 Ferrara, Italy; 3 Department of Radiology, S. Maria delle Croci Hospital, 48121 Ravenna, Italy; patrizia.cenni@auslromagna.it

**Keywords:** encephalopathy, splenial lesions, brain MRI, child

## Abstract

Reversible lesions involved in the splenium of corpus callosum (RESLES) are a rare clinic-radiological condition, whose pathogenesis could be related to infectious events (such as in mild encephalopathy with reversible splenial lesion—MERS), epilepsy or metabolic/electrolyte disorders. MERS is characterized by an acute mild encephalopathy associated with lesions in the splenium of corpus callosum on brain magnetic resonance imaging. Viral infections are commonly associated with this condition and type A influenza is the most common cause. The prognosis is generally favorable with spontaneous resolution of clinical and radiological abnormalities. We report a case report of type B influenza MERS in an 8-year-old unvaccinated girl with complete clinical and radiological recovery.

## 1. Introduction

In the literature, the presence of reversible lesions specifically involving the splenium of the corpus callosum (RESLES) has been associated with several disorders of varied origin. The availability of increasingly sophisticated magnetic resonance imaging (MRI) sequences has allowed for a better definition of this condition, which seems to constitute an entity with a favorable prognosis. RESLES has been reported secondary to acute or subacute encephalitis/encephalopathy, antiepileptic drug toxicity or withdrawal, high-altitude cerebral edema, hypoglycemia, hyponatremia or hypernatremia. Broadly speaking, the spectrum of RESLES also includes infectious events, such as in mild encephalitis/encephalopathy with a reversible splenial lesion (MERS) [1,2,3].

MERS is a clinic-radiological syndrome first described by Tada et al. in 2004 [4]. The neurologic clinical feature of RESLES, in children with MERS, is a mild encephalopathy following prodromal symptoms such as fever, cough, vomiting, diarrhea, abdominal pain and headache. The most evident neurologic symptoms are disturbance of consciousness, abnormal speech, delirious behavior, seizures, muscle weakness, ophthalmoplegia, visual hallucinations, ataxia, facial nerve and paralysis [2,3,4,5]. The pathogenesis of MERS is still not completely known. A literature review highlights that is probably due to a primary infection of the brain tissue or a complication secondary to the immune response and inflammation in infected subjects without any attributed causative agent in cerebrospinal fluid cultures. It has been suggested that transient splenium of the corpus callosum lesions likely reflect rapidly resolving intramyelinic edema or the influx of inflammatory cells and macromolecules, combined with related cytotoxic edema and hypotonic hyponatremia, which result from infection [2,3].

The presence of transient lesions involving the splenium of corpus callosum, caused by an acute brain inflammation, has been described in patients with encephalitis/encephalopathy by viruses (Rotavirus, Adenovirus, Influenza A and B, Parainfluenza, Epstein–Barr virus, Mumps virus, Herpes simplex virus, Parvovirus B-19 and Cytomegalovirus) and bacteria (Mycoplasma pneumoniae, Streptococcus pneumoniae, Salmonella and Campylobacter jejuni) [2,5,6,7].

In particular, influenza is a viral pathogen that can be an under-recognized cause of central nervous system dysfunction. Among the three influenza virus-types (A, B, and C), influenza A and B are clinically the most important, being responsible for severe epidemics in humans, and are conventionally thought to cause severe illnesses. It is known that influenza infections in children may be associated with acute onset brain dysfunction, characterized by disturbance of consciousness and abnormal behavior [6].

In MERS, brain MRI lesions may be limited to splenium (type I, ovoid shape) or extend into callosal radiations, frontoparietal subcortical white matter, to the rest of corpus callosum, and even cerebellum (type II). Clinical and radiologic outcome is generally favorable; symptoms resolved rapidly over 4–6 days, followed by complete neurological recovery. Patients with type II lesions on MRI, instead, may develop neurologic sequelae and lesions may persist on MRI for months even if their size diminishes independently of neurologic sequelae [5,8].

## 2. Case Report

An 8-year-old girl presented to our Emergency Department because of an episode of revulsion of the eyeballs and loss of strength to all four limbs with no disturbance of consciousness. The day before the symptoms occurred, she manifested dysartria, dysnomia and mental confusion lasting about one hour1 h. This symptomatology was associated with a history of 4 days of fever, apathy, asthenia, cough and headache. She had never been vaccinated. On admission she was feverish, with a normal conscious state but slurred speech. There was no evidence of focal neurological deficit. Neither meningism nor cranial nerve dysfunction were present. Laboratory values were normal. The RT-PCR performed on nasopharyngeal aspiration, nowadays considered the gold standard for respiratory pathogen detection in children, resulted positive for influenza B virus.

**Figure 1:**
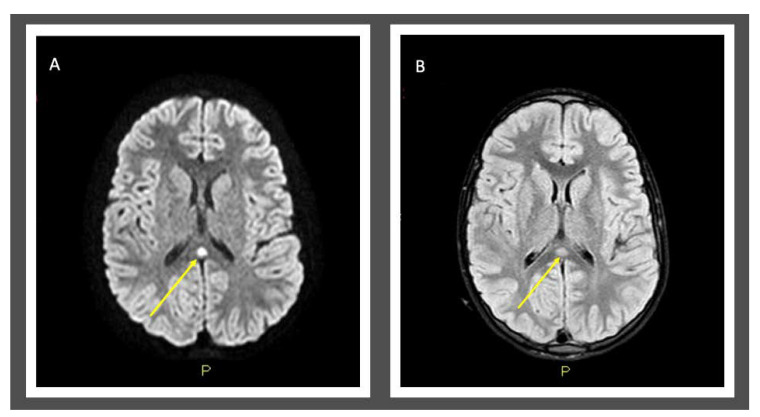
MRI of the brain (performed at day-4 of illness) showed splenial signal hyperintensity on T2-weighted sequence (**A**) and splenial restricted diffusion on diffusion weighted imaging (**B**).

The electroencephalography (EEG) report showed slow-spike theta waves bilaterally in the temporal regions, more represented on the left side. Upon brain MRI exam, we observed an acute lesion (8 mm in diameter) in the splenium of corpus callosum, hyperintense on T2-weighted images and hypointense on T1-weighted images, typical of type I MERS (Figure 1). Being the diagnosis a clear picture of MERS by type B Influenza, because of the onset of the symptoms < 48 h, an antiviral therapy with Oseltamivir (5 mg/Kg/die in 5 days) was started, with gradual resolution of the symptom. Lumbar puncture was not performed because swab positivity for influenza B and typical MRI features of the brain were diagnostic for MERS (Table 1). 

The patient presented reduced verbal communication lasting 4 days and fever for a total of 8 days. No antiepileptic therapy was required because of the absence of seizures during the hospitalization. 

The follow-up EEG report and brain MRI, performed at 30-days from complete resolution of the symptoms, were normal. The girl was seen monthly for 6 months. The evaluation of the neuropsychological development texts did not show any sequelae.

It is interesting to notice that, during the hospitalization, the patient’s twin brother presented to our attention because of an episode of visual hallucinations during fever. He also resulted positive for type B influenza. Brain MRI exam and EEG performed on him showed no lesions.

## 3. Discussion and Conclusions

RESLES is a clinical condition, still rarely described in pediatric age, and MERS is one of its most common causes [1]. The syndrome mainly affects children and young adults, and the vast majority of the cases described in the literature involve Asian and Australian children (5). Viral infections are frequently associated with this condition [2,5,7]. Although it is more frequently associated with type A influenza, MERS can be related, albeit rarely, to influenza B [5,7,8]. 

In our case MERS was a complication of an influenza B primary infection, and the outcome was favorable with complete resolution of clinical and imaging findings. The transient nature of lesions suggests that the effect of the virus on brain (including intramyelinic axonal edema, inflammatory infiltrates, oxidative stress and fluid imbalance) is reversible, and the MRI lesions may be the only detectable change in patients with usually good prognosis [3,4,5]. Complete recovery typically occurs within one month [4].

Actually it is known that there is no specific treatment for MERS. The use of antiviral therapy (in the influenza-related cases) could be effective to reduce viral expression and consequently the inflammatory response which is the first cause of the damage [6].

It is important to improve awareness of RESLES among pediatricians and radiologists. Children who present with acute encephalopathy (abnormal speech, delirious behavior, seizures, muscle weakness, ophthalmoplegia, visual hallucinations, ataxia, facial nerve and paralysis) or alterations of the state of consciousness during or immediately after influenza or other viral infections should be investigated with brain MRI exam and diffusion-weighted imaging (DWI) examinations because of the possible occurrence of MERS [3,5].

## Figures and Tables

**Table 1:** MERS Learning Points.

	Learning Points
1	MERS is rare clinic-radiological syndrome characterized by acute mild encephalopathy and brain MRI lesion evident in the splenium of corpus callosum (type I) that could extend into callosal radiations, frontoparietal subcortical white matter, to the rest of corpus callosum, and cerebellum (type II).
2	Viruses are most commonly related to MERS.
3	Prognosis is good if lesions are limited to splenium of corpus callosum.
4	Consider MERS in children with clinical signs of encephalopathy (neurologic symptoms or alterations of the state of consciousness) during a viral infectious event and investigate it with brain MRI exam and DWI examinations.

## Data Availability

Not applicable.

## References

[1] García-Moncó J.C., Cortina I.E., Ferreira E., Martínez A., Ruiz L., Cabrera A., Beldarrain M.G. (2011). Reversible Splenial Lesion Syndrome (RESLES): What’s in a Name?. J. Neuroimaging.

[2] Takanashi J.-I., Tada H., Maeda M., Suzuki M., Terada H., Barkovich A.J. (2009). Encephalopathy with a reversible splenial lesion is associated with hyponatremia. Brain Dev..

[3] Zhang S., Ma Y., Feng J. (2015). Clinicoradiological spectrum of reversible splenial lesion syndrome (RESLES) in adults: A retrospective study of a rare entity. Medicine.

[4] Tada H., Takanashi J., Barkovich A.J., Oba H., Maeda M., Tsukahara H., Suzuki M., Yamamoto T., Shimono T., Ichiyama T. (2004). Clinically mild encephalitis/encephalopathy with a reversible splenial lesion. Neurology.

[5] Chen W.-X., Liu H., Yang S.-D., Zeng S.-H., Gao Y.-Y., Du Z.-H., Li X.-J., Lin H.-S., Liang H.-C., Mai J. (2016). Reversible splenial lesion syndrome in children: Retrospective study and summary of case series. Brain Dev..

[6] Fumarola A., Ricciardelli P., Guiducci C., Turlà G., Cenni P., Marchetti F. (2019). Encephalitis by type B influenza: A pediatric clinical case and literature review. Recenti Prog. Med..

[7] Yıldız A.E., Genç H.M., Gürkaş E., Ünlü H.A., Öncel I.H., Güven A. (2018). Mild encephalitis/encephalopathy with a reversible splenial lesion in children. Diagn. Interv. Radiol..

[8] Vanderschueren G., Schotsmans K., Maréchal E., Crols R. (2018). Mild encephalitis with reversible splenial (MERS) lesion syndrome due to influenza B virus. Pract. Neurol..

